# Towards a Universal Translator: Decoding the PTMs That Regulate Orthoflavivirus Infection

**DOI:** 10.3390/v17020287

**Published:** 2025-02-19

**Authors:** Hannah M. Schmidt, Stacy M. Horner

**Affiliations:** 1 Department of Molecular Genetics and Microbiology, Duke University School of Medicine, Durham, NC 27710, USA; hannah.schmidt@duke.edu; 2 Department of Integrative Immunobiology, Duke University School of Medicine, Durham, NC 27710, USA; 3 Department of Medicine, Duke University School of Medicine, Durham, NC 27710, USA

**Keywords:** post-translational modifications, flavivirus, ubiquitin

## Abstract

Post-translational modifications (PTMs) serve as critical regulators of protein function across biological systems, including during viral infection. For orthoflaviviruses, including human pathogens like dengue, Zika, and West Nile viruses, PTMs on viral proteins regulate multiple aspects of the viral lifecycle and pathogenesis. Here, we review the mechanisms by which PTMs regulate orthoflavivirus infection in both vertebrate and arthropod hosts. We examine how ubiquitination and glycosylation on the viral envelope proteins facilitate viral entry and how phosphorylation, SUMOylation, and acetylation on non-structural proteins modulate viral RNA replication. Additionally, we describe how PTMs on viral structural proteins dynamically regulate viral assembly and egress. We also describe how PTMs can influence tissue tropism and host-specific pathogenesis, with some modifications showing divergent functions between arthropod vectors and vertebrate hosts, and how the host antiviral response can trigger specific PTMs on viral proteins to restrict infection, highlighting PTMs as key mediators of host-pathogen interactions. While significant progress has been made in identifying PTMs on viral proteins, many questions remain about their temporal dynamics, mechanisms of action, and conservation across the orthoflavivirus genus. Understanding how PTMs regulate orthoflavivirus infection may reveal new therapeutic strategies, particularly given recent advances in targeting specific protein modifications for disease treatment.

## 1. Introduction

Post-translational modifications (PTMs) provide reversible control of the processes that regulate viral infection. In general, PTMs modulate protein function by altering the stability, activity, interaction partners, and subcellular location of the modified protein. Regulation of protein function by PTMs ultimately plays critical roles in mediating cellular processes, such as the cell cycle, transcription, signal transduction, and organelle remodeling. Proteins can be post-translationally modified through different processes, including proteolytic processing, the addition of chemical groups such as phosphorylation or acetylation, the addition of oligosaccharide groups through glycosylation, and the addition of small proteins such as ubiquitin or ubiquitin-like modifiers (Table 1). One enzyme can add these PTMs, such as a kinase in the case of phosphorylation, or multiple enzymes that function in an enzymatic cascade can add PTMs, such as in the conjugation pathways for ubiquitin and ubiquitin-like modifications. The cellular machinery that regulates the addition of PTMs can be co-opted by RNA viruses to expand their regulatory potential.

Orthoflaviviruses are a genus of positive-sense RNA viruses that includes notable pathogens such as dengue virus (DENV), Japanese Encephalitis virus (JEV), tick-borne encephalitis virus (TBEV), West Nile virus (WNV), yellow fever virus (YFV), and Zika virus (ZIKV), all of which represent significant human health burdens [1]. These arthropod-transmitted viruses put billions of people at risk annually with differing clinical presentations, ranging from asymptomatic infection or mild illness to severe diseases that consist of hemorrhagic fever, encephalitis, or hepatitis. Despite their distinct clinical presentations, orthoflaviruses share similar molecular mechanisms, underscoring the need for a comprehensive understanding of the molecular processes that regulate their infection. Due to their limited genome size, orthoflaviviruses often utilize host proteins, including enzymes that catalyze PTMs, to regulate their lifecycles. These viral lifecycle stages must be tightly coordinated to organize infection in space and time, and PTMs of viral proteins can help facilitate this coordination. Similarly, to function on cellular proteins, PTMs on viral proteins can regulate infection through modulation of protein stability, enzymatic activity, and protein-protein interactions. A diverse complement of PTMs on orthoflaviviral proteins have been described, including phosphorylation, ubiquitination, SUMOylation, acetylation, and glycosylation (also reviewed in [2,3]; Table 2). These modifications have been identified through direct methods, such as immunoprecipitation of viral proteins or PTMs followed by mass spectrometry, or through indirect methods, such as examining the role of a cellular post-translational modifier in orthoflaviviral infection. Importantly, PTMs play a key role in regulating orthoflaviviral infection in both human and arthropod hosts. This review will focus on the mechanisms by which PTMs, specifically the addition of chemical and protein groups to orthoflavivirus proteins, regulate key lifecycle stages and pathogenesis and contextualize these studies in the framework of the lifecycle and pathogenesis of orthoflaviviruses.

**Table 1:** General description of PTMs regulating orthoflaviviruses.

Classifications	Modification	Description	Target Residues	Enzymes
Carbohydrates	N-linked Glycosylation	The covalent addition of carbohydrate chains to protein molecules [4].	Asparagine (N)	Glycosyltranferases (addition), glycosidases (removal)
	ADP-ribosylation	Covalent addition of ADP-ribose to target proteins [5].	Serine (S), Threonine (T), Tyrosine (Y), Glutamate (E), Aspartate (D), Cysteine (C), Arginine (R), Lysine (K) and Histidine (H)	ADP-ribosyltransferases
Chemical groups	Phosphorylation	Addition of a phosphate group to amino acid side chains of a target protein [6].	Serine (S), Threonine (T), or Tyrosine (Y)	Kinases (addition), phosphatases (removal)
	Lysine acetylation	Covalent attachment of an acetyl group onto target proteins [7].	Lysines (K)	Lysine acetyltransferases
Ubiquitin-like modification	Ubiquitination	Covalent attachment of ubiquitin onto target proteins can be degradative (K48) or non-degradative (K63), depending on the linkage [8].	Lysines (K)	Ub-activating enzymes (Ub-E1), Ub-conjugating enzymes (Ub-E2), Ub-ligating enzymes (Ub-E3)
	SUMOylation	Covalent attachment of small ubiquitin-like modifier (SUMO) onto target proteins [9].	Lysines (K)	SUMO-activating enzyme (SAE1/SAE2), SUMO-conjugating enzyme (Ubc9), SUMO-ligating enzymes (SUMO-E3)
	ISGylation	Covalent attachment of the ubiquitin-like protein interferon-stimulated gene 15 (ISG15) onto target proteins [10].	Lysines (K)	ISG15-activating enzyme (UBA7), ISG15-conjugating enzyme (UBE2L6), ISG15-ligating enzymes (ISG15-E3)

## 2. PTMs on Viral Proteins Regulate Orthoflaviviral Entry and Genome Uncoating

The entry and uncoating of orthoflaviviruses involve multiple steps regulated by PTMs, particularly ubiquitination and glycosylation. Orthoflaviviral infection begins when the viral E proteins on the virion surface bind to attachment factors and receptors on the cell surface, facilitating viral attachment and subsequent cell entry [33]. Orthoflaviviruses have a broad range of cellular attachment factors and receptors, including TIM and TAM family receptors, heparin sulfates, DC-SIGN, and DC-SIGNR [34,35]. Following attachment, virions enter the cell via clathrin-mediated endocytosis. The viral membrane then fuses with the endosomal membrane through pH-mediated conformational changes of the E protein during endosome acidification, releasing the nucleocapsid, consisting of the viral Capsid proteins bound to the viral RNA genome, into the cytoplasm [33]. Subsequently, the nucleocapsid disassembles, and Capsid is released from the viral RNA, resulting in free, “uncoated” viral RNA molecules that can be translated [36]. Together, these steps—from receptor-mediated entry through endocytosis to pH-dependent membrane fusion and nucleocapsid disassembly—constitute the early stages of orthoflavivirus infection that are regulated by PTMs.

**Figure 1:**
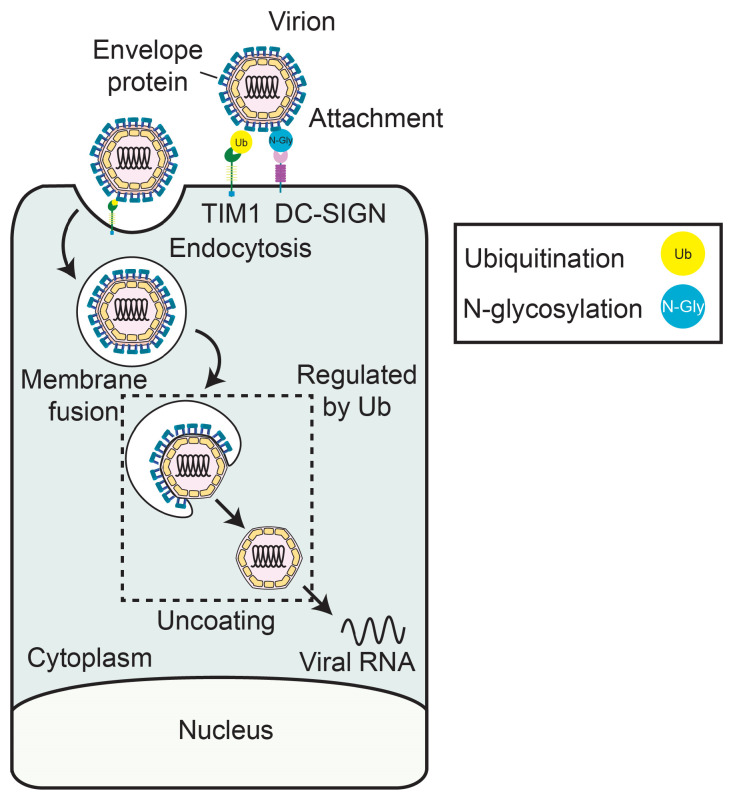
**Post-translational modifications of orthoflavivirus proteins regulate virus entry and genome uncoating.** A diagram of the orthoflavivirus lifecycle from attachment through genome uncoating. Post-translational modifications, such as glycosylation and ubiquitination, regulate orthoflaviviral attachment and genome uncoating.

During the entry process, PTMs on the orthoflavivirus E protein can regulate viral attachment and receptor interactions with the virion. For example, glycosylation of the WNV, JEV, ZIKV, and DENV E protein, which has been extensively reviewed [2], promotes interaction with a viral attachment factor, DC-SIGN (Figure 1). In addition to glycosylation, in ZIKV, K63-linked ubiquitination of the viral E protein at a specific lysine residue (K38) by TRIM7 promotes viral attachment by facilitating binding between E and one of the orthoflavivirus receptor protein, TIM1 (Figure 1) [13]. This ubiquitination event promotes entry into human cells and most mouse tissues, including the brain and reproductive organs, but not into mosquito cells, highlighting host-specific differences in receptor usage [13]. These findings demonstrate that both glycosylation and ubiquitination of E protein regulate viral entry through distinct mechanisms that can vary across hosts.

Ubiquitination also plays an essential role in uncoating, facilitating either the release of the nucleocapsid from the endosome or the uncoating of the viral RNA, although the exact mechanisms of how ubiquitination regulates these processes are unknown. This is the case during DENV infection, where ubiquitination is required for DENV genome uncoating [37]. The protein target of this ubiquitination is unclear. Specifically, inhibition of the ubiquitin E1 enzyme (UBA1) prevents initial translation of the DENV genome only during infection but not when viral RNA is directly transfected into cells [37]. While this result suggests that ubiquitin could regulate genome uncoating, experiments that measure uncoating by measuring translation of the incoming viral RNA found that mutation of all capsid lysine residues to residues that prevent ubiquitination did not affect viral RNA translation, indicating that ubiquitination of capsid is specifically dispensable for genome uncoating. These results implicate another protein as the ubiquitinated protein that facilitates genome uncoating, perhaps by regulating its exit from the endosome, membrane fusion, or release of viral RNA from nucleocapsids (Figure 1). Ubiquitination can also promote genome uncoating of YFV and JEV, although, similar to DENV, the target of ubiquitination in these infections remains unclear [38,39]. These findings reveal examples of how ubiquitination and glycosylation can regulate orthoflavivirus entry and uncoating, though mechanistic details remain to be elucidated.

## 3. PTMs on Viral Proteins Regulate Viral Replication and Membrane Reorganization

Orthoflavivirus replication and membrane reorganization are regulated by multiple PTMs. Following genome uncoating, the orthoflaviviral RNA genome is translated by ribosomes on the ER membrane [40]. The genome is translated as a single polyprotein and co-translationally inserted into the ER, where it is cleaved by the viral NS2B-NS3 protease, along with host proteases, into the individual viral proteins [41]. These proteins include non-structural proteins that mediate viral replication and ER membrane reorganization to facilitate infection. Then, viral genome replication is catalyzed by NS5. This protein encodes several enzymatic functions required for replication, including an N-terminal domain important for 5′ RNA capping and 2′-O-methylation and a C-terminal domain that encodes the RNA-dependent RNA polymerase domain (RdRp). The RdRp of NS5 copies the positive-strand viral RNA genome into a negative-strand RNA intermediate. NS5 then synthesizes additional positive-strand genomes from the negative-strand intermediate and facilitates 5′ RNA capping and 2′-O-methylation. In addition to NS5, other non-structural proteins, including the viral helicase NS3, are also required for orthoflavivirus genome replication [41,42]. For efficient genome replication of orthoflaviviruses to occur, viral replication factors, such as viral proteins and RNA, are concentrated in reorganized ER membrane structures called ER invaginations. These ER invaginations are formed through the actions of several viral proteins, including NS1, the NS4A-2K-4B precursor, and processed NS4A and NS4B [42,43]. In addition to forming ER invaginations that serve as replication organelles during infection, viral proteins also induce the formation of convoluted membranes, which are dense ER structures that contain the viral proteins NS3, NS4B, and NS2B but no viral RNA [24,44,45]. The exact roles of convoluted membranes in infection are unknown, but evidence suggests that they may promote the degradation of non-structural proteins present in excess to promote infection [24]. Flavivirus replication and ER membrane reorganization have been reviewed extensively [41,42,46]. Orthoflavivirus proteins involved in both replication and ER membrane reorganization are post-translationally modified.

**Figure 2:**
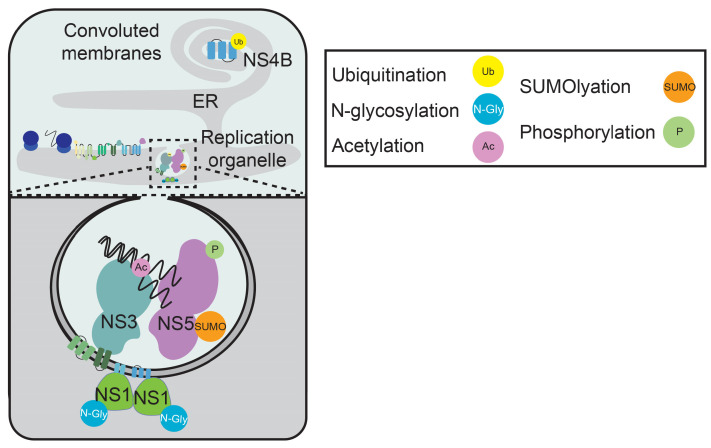
**Post-translational modification of orthoflavivirus proteins virus regulates viral replication and membrane reorganization.** A diagram of orthoflavivirus-induced ER rearrangements, with a zoomed-in diagram of replication sites at ER invaginations. Several different post-translational modifications on viral proteins regulate RNA replication and ER rearrangements.

Orthoflavivirus RNA replication can be regulated by PTMs of several viral proteins, including NS5 and NS3. NS5 is post-translationally modified by phosphorylation and SUMOylation to promote its stability or function, thereby impacting replication. For example, the DENV NS5 protein is phosphorylated on a threonine residue (T449) within its RdRp domain in mammalian cells, likely by protein kinase G (Figure 2) [29]. Intriguingly, this residue is conserved as either threonine or serine in mosquito-borne orthoflaviviruses, suggesting that phosphorylation of this residue is broadly required by these viruses. Importantly, loss of phosphorylation at this position by mutation of the threonine to the conserved histidine (T449H) present in the NS5 of tick-borne orthoflaviviruses abrogates viral replication in mammalian cells, suggesting differing replication mechanisms between mosquito- and tick-borne orthoflaviviruses in these cells. In YFV, the N-terminal domain of the NS5 protein is phosphorylated at S56 [30]. Loss of phosphorylation at this residue by mutation to alanine (S56A) or the phosphomimetic mutation S56D both inhibit 2′-O methylation of YFV RNA and result in attenuated replication. Due to the high degree of conservation of a serine at this site in NS5, phosphorylation reversibility at this site may be important for viral infection. In addition to phosphorylation, SUMOylation of orthoflaviviral NS5 has been described (Figure 2) [31]. During DENV infection, NS5 is SUMOylated, and loss of SUMOylation destabilizes NS5 and impairs RNA replication. In summary, the orthoflaviviral protein NS5 is post-translationally modified to promote its multiple roles in RNA replication and its stability, but the enzymes catalyzing some of these PTMs remain to be identified. In addition to NS5, the orthoflaviviral NS3 protein is post-translationally modified. Specifically, the helicase domain of ZIKV NS3 is acetylated at K389 [25]. This acetylation is mediated by the acetyltransferase KAT5γ, which promotes viral infection. Mechanistically, mutational studies reveal that K389 acetylation tunes RNA binding to an optimal level that promotes helicase activity and viral RNA replication. Acetylation at NS3 K389 is conserved in WNV, and the NS3 proteins of DENV and YFV are also acetylated, although the site of acetylation on DENV and YFV NS3 proteins is unknown. Together, these studies show that orthoflaviviral RNA replication is regulated by PTMs on viral proteins to promote their stability or enzymatic functions.

Multiple orthoflaviviral proteins involved in ER membrane remodeling are regulated by glycosylation and ubiquitination to promote viral infection. For example, the orthoflavivirus NS1 protein is glycosylated on N130 and N207, facilitating replication [17,47,48], and mutational studies reveal that, in ZIKV, these glycosylation sites may contribute to NS1-mediated ER rearrangements (Figure 2) [16]. Other orthoflaviviral proteins with roles in ER rearrangements, NS4A and NS4B, have been described to be ubiquitinated. Specifically, NS4A from either DENV or JEV can be ubiquitinated at K80 or K79, respectively, by the cellular E3 ubiquitin ligase HRD1, inducing NS4A degradation in mammalian or mosquito cells [28]. Interestingly, this NS4A ubiquitination event helps degrade excess NS4A to promote viral infection. Others have also shown that JEV NS4A, as well as NS2A and NS4B, are ubiquitinated and subsequently degraded [24]. This study found that the cellular protein Derlin2 was required for this ubiquitination and that Derlin2 promotes infection, suggesting that the ubiquitination and degradation of these viral proteins promotes infection. In later time points of infection, these viral proteins are localized to convoluted membranes (Figure 2), and it may be that their degradation as a result of ubiquitination prevents cellular toxicity. In summary, these studies show that PTMs on viral proteins contribute to orthoflavivirus-induced membrane rearrangements and may facilitate protein degradation at convoluted membranes.

## 4. PTMs on Viral Proteins Regulate Viral Assembly and Egress

PTMs regulate the function of multiple orthoflavivirus proteins involved in assembly and egress. To begin viral assembly, newly synthesized positive-sense strand orthoflaviviral RNA genomes are brought to sites of viral assembly on the ER through interaction with the viral NS2A protein [49,50]. Concurrently, NS2A interaction with both the prM-E precursor protein and the NS2B-NS3 protease facilitates proteolytic processing of the Capsid-prM-E precursor protein complex to release the Capsid protein [51,52]. The genomic RNA associates with the processed Capsid to form the viral nucleocapsid, which then buds through the ER to form an immature virion containing prM and E [1]. The virion then traffics through the secretory pathway, with additional prM maturation occurring in the Golgi to generate mature infectious virions, which are then secreted from the cell [52]. PTMs on both Capsid and E can regulate the production of infectious virions.

**Figure 3:**
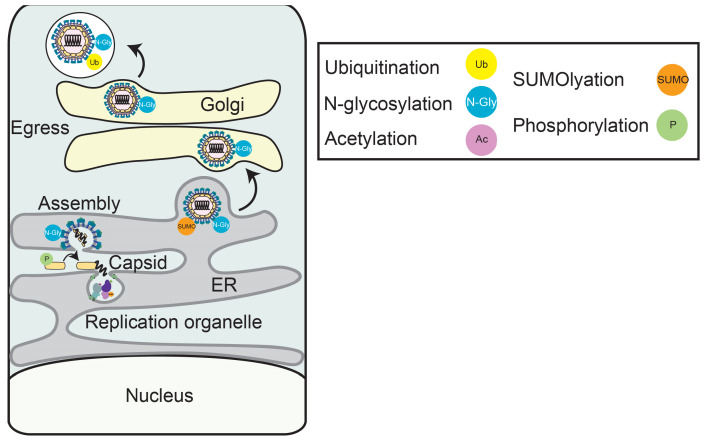
**Post-translational modifications of orthoflavivirus proteins regulate viral assembly and egress.** A diagram of the orthoflavivirus lifecycle from assembly through egress. Several post-translational modifications on viral proteins regulate assembly and egress.

Phosphorylation and SUMOylation of the orthoflavivirus structural proteins Capsid and E promote viral assembly and egress. For example, the phosphorylation of Capsid protein dynamically regulates orthoflaviviral assembly. During WNV infection, Capsid is phosphorylated, likely by protein kinase C, on specific serine and threonine residues [11,53]. This Capsid phosphorylation decreases at later time points during infection, and in vitro studies reveal that these phosphorylation events limit Capsid binding to viral RNA, which is essential for nucleocapsid assembly. Together, this supports a model where Capsid is dephosphorylated during the late stages of infection to promote nucleocapsid assembly (Figure 3). In addition to regulating viral assembly, PTMs on viral structural proteins promote orthoflaviviral egress. For example, SUMOylation of the DENV E protein is suggested to promote viral egress in mosquito cells [14]. While the exact mechanisms for how SUMOylation may regulate viral egress is not yet clear, mutations that abrogate SUMOylation do decrease pseudovirus production, and loss of the E2 enzyme of SUMOylation, Ubc9, inhibits viral infection in mosquito cells, revealing that viral egress does require SUMOylation (Figure 3) [14,54]. Together, these studies show that orthoflaviviral structural protein PTMs regulate nucleocapsid assembly and egress.

## 5. Host Responses Can Facilitate PTMs on Viral Proteins

Host cells can also induce PTMs on orthoflaviviral proteins as part of the host defense system to restrict infection and promote cell survival. Orthoflavivirus infection induces interferons and interferon-stimulated genes (ISGs). Some of these ISGs restrict orthoflaviviral infection by inducing PTMs on viral proteins. For example, both ZIKV NS1 and NS3 proteins are ADP-ribosylated by the ISG PARP12, an ADP-ribosyltransferase, to restrict infection [21]. Interestingly, PARP12 over-expression also results in K48-linked ubiquitination on NS1 and NS3 and their proteasome-mediated degradation. This degradation is dependent on PARP12 ADP-ribosylation activity. This supports a model where PARP12 ADP-ribosylation on ZIKV NS1 and NS3 facilitates interaction with an unknown ubiquitin E3 ligase to induce K48-linked ubiquitination and proteosome-mediated degradation of ZIKV NS1 and NS3, although the sites for ubiquitination and ADP-ribosylation on NS1 and NS3 are unknown. In DENV, the viral NS3 protein is modified by the ISG TRIM69, an E3 ubiquitin ligase, to restrict infection [26]. Mutational analyses reveal that TRIM69 ubiquitinates DENV NS3 at K104, leading to its degradation and thus inhibiting replication. Similarly, the ZIKV NS1 and NS3 proteins are ubiquitinated by the ISG and E3 ligase TRIM22 and then subsequently degraded, which inhibits ZIKV infection [22]. Further, the NS2B-NS3 protease of the tick-borne orthoflavivirus Langat virus (LGTV) is modified by the ISG TRIM5α, an E3 ubiquitin ligase [27]. Beyond LGTV, TRIM5α restricts the infection of multiple tick-borne, but not mosquito-borne, orthoflaviviruses by facilitating K48-linked ubiquitination and subsequent degradation of the viral NS2B-NS3. DENV NS3 and NS5 have also been suggested to be post-translationally modified by ISG15, a ubiquitin-like post-translational modification conjugated to lysine residues [55]. While over-expression of the ISGylation machinery restricts DENV infectious particle production, neither the exact NS3 and NS5 residues modified, the E3 ligase of ISGylation involved, nor the mechanism of action are known. These studies highlight the PTMs on orthoflavivirus proteins induced by the antiviral host defense system.

Post-translational modifiers not directly induced by the antiviral host defense system can also provide protection against orthoflavivirus infection by acting on viral proteins. The WNV Capsid protein is modified by MKRN1, a constitutively expressed ubiquitin E3 ligase, which restricts WNV infection [12]. MKRN1 catalyzes K48-linked ubiquitination of Capsid at specific residues (K101, K103, and K104), which facilitates proteasomal degradation of Capsid, suggesting that MKRN1 restricts infection by inducing Capsid degradation. In addition to direct PTM of orthoflaviviral proteins, viruses can manipulate the host response by altering PTMs on host proteins through several mechanisms. These include facilitating proteolytic cleavage of host proteins, recruiting post-translational modifier machinery to host proteins, or binding host proteins to prevent their modification [44,56,57,58,59,60]. Manipulation of host protein PTMs by viruses is extensively reviewed in these sources [61,62,63]. PTMs on both viral and host proteins regulate the antagonistic interplay between viral survival strategies and host defense mechanisms at the virus-host interface. Taken together, the above studies demonstrate that the manipulation of PTMs on orthoflavivirus proteins can have antiviral functions during infection.

## 6. PTMs on Viral Proteins Regulate Infection in Arthropods

PTMs on orthoflaviviral proteins also have roles in regulating pathogenesis in the arthropod vector. Medically relevant orthoflaviviruses are primarily spread to people from arthropods. Orthoflaviviruses must overcome barriers to infection in arthropods and vertebrates to maintain a transmission cycle between multiple hosts [64]. For mosquito-borne orthoflaviviruses in order to transmit from the arthropod vector, the virus must establish infection in the midgut after a bloodmeal, followed by dissemination from the midgut to other tissues like the salivary glands to facilitate new infection [65]. Glycosylation of ZIKV E protein promotes infection in mosquitos [15,66]. Mechanistically, glycosylation of ZIKV E protein at N154 facilitates viral infection through the midgut by inhibiting the production of reactive oxygen species, which are important regulators of the mosquito antiviral response [15]. Orthoflaviviruses can have species-specific host factor requirements. For example, ZIKV NS1 ubiquitination by WWP2 or its mosquito homolog Su(dx) can be observed in both human and mosquito cells [23]. In human cells, WWP2 is antiviral, facilitating NS1 K63- and K48-linked ubiquitination at K265 and K284, respectively, which promotes NS1 proteasomal degradation. However, in mosquito cells, Su(dx) promotes viral infection and induces K63-linked ubiquitination of NS1 at K265, indicating that NS1 ubiquitination likely promotes ZIKV infection in mosquito cells. Maintenance of a lysine position at K265 in ZIKV populations, which allows for pro-viral ubiquitination in mosquitos and antiviral ubiquitination in human cells, demonstrates the evolutionary trade-offs associated with transmission between multiple host species. Together, these studies demonstrate that PTMs on orthoflaviviral proteins can serve species-specific functions during infection.

## 7. PTMs on Viral Proteins Regulate Pathogenesis in Vertebrates

PTMs on orthoflaviviral proteins have roles in regulating orthoflaviviral pathogenesis by altering tissue tropism and interactions with the host immune system. Orthoflaviviruses have a wide range of disease manifestations, from completely asymptomatic infection to hemorrhagic fever, severe congenital defects, or encephalitis [1]. The underlying causes of this variation in disease severity are not fully understood but can be partially attributed to prior infection status, differences in host response, and tissue and cellular tropism of the orthoflavivirus. PTMs of orthoflaviviruses, particularly glycosylation of E and NS1 proteins, impact disease severity in a mouse model. Mutations in ZIKV and WNV virus that prevent glycosylation of E lead to lower viremia and reduced mortality in mice [66,67,68]. Interestingly, ZIKV lacking E glycosylation at N154 results in decreased mortality compared to the parental ZIKV strain when injected subcutaneously but not when injected intracranially, suggesting that the E glycosylation of ZIKV facilitates bypassing the blood-brain barrier [66,67]. In addition to E glycosylation, loss of NS1 glycosylation through mutation of the glycosylation site(s) in NS1 of WNV and ZIKV also results in reduced viremia and mortality in mice [18,19]. DENV and YFV lacking NS1 glycosylation also have reduced mortality in mice, although viremia was not measured [17,20]. In addition to regulating mortality, glycosylation of E also impacts tissue tropism. In infected mice, ZIKV lacking E glycosylation at N154 has decreased infection in the brain and eyes but not in all tissues measured [67]. Similarly, the ubiquitination of ZIKV E impacts tissue tropism in mice, with ZIKV lacking E ubiquitination at K34 having decreased viral load in select tissues, most significantly the brain, lung, and testes [13]. While these studies in mice have significantly advanced our understanding of how orthoflavivirus PTMs may regulate pathogenesis, differences in PTMs between mouse and human cells have been described [32]. Consequently, these findings may not fully capture the role of PTMs in viral pathogenesis in humans. Together, these studies demonstrate how PTMs on orthoflaviviral proteins can regulate viral pathogenesis by mediating virulence and tissue tropism.

## 8. Discussion

In this review, we have described how diverse PTMs on orthoflaviviral proteins regulate infection and pathogenesis in both vertebrate and arthropod hosts. PTMs control key viral processes by altering protein stability, modulating enzymatic activity, and orchestrating protein-protein interactions. We have shown how ubiquitination and glycosylation facilitate viral entry and genome uncoating while phosphorylation and SUMOylation of non-structural proteins, like NS5 and NS3, regulate viral replication. Additionally, we have described how PTMs of structural proteins coordinate viral assembly and egress and how PTMs of orthoflaviviral proteins influence tissue tropism and pathogenesis. Notably, some PTMs serve different functions between arthropod vectors and vertebrate hosts, highlighting their role in host adaptation.

While the studies we describe in this review have expanded our knowledge of the PTMs present on viral proteins during infection, our understanding of the full scope of PTMs regulating orthoflaviviral proteins and their mechanism of action remains incomplete. In particular, the role of several ubiquitin-like modifications in orthoflaviviral infection, such as NEDD8, FAT10, and UFM1, have yet to be described, although they do have roles in other viral infections [9,69,70]. Additionally, although the roles of many PTMs on viral proteins in particular lifecycle stages have been described, there is much we do not know about how the PTMs are regulated. Specifically, the timing of when in the viral lifecycle these modifications are added to viral proteins has not been explored. Understanding this timing is important to learn how PTMs could be dynamic regulators of orthoflaviviral infection. Indeed, as many of these modifications are reversible, future studies focusing on the dynamics of these modifications during orthoflaviviral infection are important. For example, the acetylation of NS3 inhibits RNA binding, but mutations in the virus that prevent NS3 acetylation inhibit viral infection [25], suggesting that the timing and reversibility of this modification may be an important regulator of NS3 function during infection. Thus, while many PTMs on orthoflaviviral proteins have been described, the mechanistic details about the addition and removal of these PTMs during infection remain largely unexplored.

In addition to PTM dynamics, there is much to be discovered about potential crosstalk between modifications on viral proteins, especially since a diverse set of PTMs can be added to lysine residues [71]. Some viral proteins have been described to have several PTMs. For example, the ADP-ribosylation of ZIKV NS1 and NS3 seems to also promote their K48-linked ubiquitination [21]. In this example, these modifications appear to function cooperatively. In other cases, modification dynamics have not been explored, and thus, for those PTMs, it is unclear whether specific PTMs compete or occur on different peptides of the viral protein. Further, it remains unknown if distinct polypeptides of a viral protein may have independent functions depending on the modification. It is also largely unknown what proportion of the viral protein targets are modified. While some modifications, such as glycosylation, seem to modify a majority of the target polypeptides, as seen by overall notable differences in protein size, many modifications are likely only on a small fraction of the target polypeptides, consistent with the proportions of modified cellular proteins [72]. As such, quantitative methods that can profile a diverse set of PTMs will need to be employed to address PTM dynamics and crosstalk on viral proteins.

PTM conservation across different orthoflavivirus species and hosts also remains a largely open question. While many modifications have been characterized in one species of orthoflavivirus, phylogenetic analysis often reveals that the modified residue(s) are conserved across a subset of species in the genus. For example, the T449 phosphorylation site of NS5 was identified during DENV infection, but excitingly, this site is conserved across mosquito-borne orthoflaviviruses [29]. This suggests that other genetically related orthoflaviviruses have the potential to be modified and regulated in a similar manner. In many cases, the full scope of orthoflaviviruses regulated by a specific modification is unknown, and it is often still unclear if the function or presence of a specific PTM on an orthoflavivirus protein is unique or conserved. In addition to our limited understanding of which orthoflaviviruses are regulated by each modification, there is still much we do not know about the cell type specificity of the described PTMs. Some described PTMs on viral proteins, such as ZIKV E ubiquitination [13], affect infection differently in multiple tissues, indicating that these PTMs may regulate infection in specific cell types. Additionally, the role of many described PTMs in regulating infection in the arthropod vector is unclear compared to the human host, even though homologs of the PTM enzymes often exist. Thus, it remains an open question if many of the modifications that regulate infection in human cells also regulate infection in arthropods. Interestingly, some modifications do serve different roles in arthropods versus humans. For example, ubiquitination on ZIKV NS1, which is anti-viral in human cells but pro-viral in mosquito cells [23], suggests evolutionary trade-offs in host-switching and highlights the complex interplay between viral proteins and host cellular mechanisms.

Studies of viral PTMs have expanded our mechanistic understanding of the regulation of orthoflavivirus lifecycles. However, significant questions remain about temporal dynamics and mechanisms of action of PTMs. The stoichiometry, reversibility, and crosstalk between different modifications on orthoflaviviral proteins are largely unexplored. Additionally, the conservation of PTMs across different orthoflaviviruses and their functions in arthropod versus vertebrate hosts requires further investigation. Understanding these aspects could reveal new therapeutic opportunities, particularly given recent advances in targeting specific PTMs for disease treatment. For example, chemical inducers of proximity that direct PTM enzymes to specific viral proteins represent a promising antiviral strategy, with similar approaches already advancing in clinical trials for cancer therapy [73,74]. Expanding our understanding of PTM dynamics during orthoflavivirus infection could enable the development of such targeted therapeutic approaches while providing fundamental insights into viral regulation.

## Figures and Tables

**Table 2:** PTMs on orthoflaviviral proteins.

Viral Protein	Virus (Residues)	Modification	Host	Pro/Anti Viral	Mechanism
Capsid	WNV (S26, 36, 83, 99 and T100)	Phosphorylation	Human	Anti	Phosphorylation of Capsid dynamically regulates Capsid-RNA binding [11].
	WNV (K101, 103, 104)	Ubiquitination	Human	Anti	Ubiquitination by MKRN1 facilitates the degradation of Capsid [12].
Envelope	DENV (N67), JEV (N154), WNV (N154), ZIKV (N154)	Glycosylation	Human	Pro	Facilitates binding to DC-SIGN and DC-SIGNR and contributes to viral pathogenesis in a mouse model (WNV and ZIKV) [2].
	ZIKV (K38)	K-63 linked Ubiquitination	Human	Pro	Facilitates binding to TIM1 and contributes to tissue tropism in a mouse model [13].
	DENV	SUMOylation	Mosquito	Pro	Proposed to facilitate viral egress [14].
	ZIKV (N154)	Glycosylation	Mosquito	Pro	Facilitates midgut invasion in the mosquito by inhibiting ROS [15].
NS1	ZIKV (N130 and N207)	Glycosylation	Human	Pro	Glycosylation of NS1 facilitates ER rearrangements [16].
	DENV (N130, N207), WNV (N130, N175, N207), YFV (N130, N208), ZIKV (N130, N207)	Glycosylation	Mouse model	Pro	Contributes to viral pathogenesis in a mouse model [17,18,19,20].
	ZIKV	ADP-ribosylation	Human	Anti	Modification by PARP12 facilitates subsequent ubiquitination and degradation of NS1 [21].
	ZIKV	Ubiquitination	Human	Anti	Ubiquitination by TRIM22 facilitates the degradation of NS1 [22].
	ZIKV (K265, K284)	K63-linked and K48-linked ubiquitination, respectively	Human	Anti	Ubiquitination by WWP2 facilitates the degradation of NS1 [23].
	ZIKV (K265)	K-63-linked ubiquitination	Mosquito	Pro	Unclear mechanism [23].
NS2B	JEV	Ubiquitination	Human	Pro	Facilitates the degradation of excess NS2B [24].
NS3	ZIKV (K389), WNV (K389), DENV, YFV	Acetylation	Human	Pro	Acetylation by KAT5γ decreases RNA binding, and loss of acetylation or irreversible acetylation decreases helicase activity [25].
	DENV (K104)	Ubiquitination	Human	Anti	Ubiquitination of DENV NS3 by the ISG TRIM69 promotes NS3 degradation [26].
	ZIKV	ADP-ribosylation	Human	Anti	Modification by PARP12 facilitates subsequent ubiquitination and degradation of NS3 [21].
	ZIKV	Ubiquitination	Human	Anti	Ubiquitination by the ISG TRIM 22 facilitates the degradation of NS3 [22].
	LGTV	Ubiquitination	Human	Anti	Ubiquitination by the ISG TRIM5α facilitates the degradation of NS2B-NS3 protease complex [27].
NS4A	DENV (K80), JEV (K79)	Ubiquitination	Mosquitos, human	Pro	Facilitates the degradation of excess NS4A [28].
	JEV	Ubiquitination	Human	Pro	Facilitates the degradation of excess NS4A [24].
NS4B	JEV	Ubiquitination	Human	Pro	Facilitates the degradation of excess NS4B [24].
NS5	DENV (T449), YFV	Phosphorylation	Human	Pro	Facilitates viral RNA replication [29].
	YFV (S56)	Phosphorylation	Human	Pro	Phosphorylation decreases 2′O methylation of viral RNA, and the loss of phosphorylation or a phosphomimetic mutation results in attenuated viruses [30].
	DENV	SUMOylation	Human	Pro	SUMOylation promotes stability of NS5 [31].
	YFV	Ubiquitination	Human	Pro	Ubiquitination promotes YFV NS5 and STAT2 binding to facilitate innate immune evasion [32].
